# Discovery and Development of Novel GSK‐3β Inhibitor for the management of Alzheimer's disease

**DOI:** 10.1002/alz70859_102577

**Published:** 2025-12-25

**Authors:** Samanta Gambhir, Manjinder Singh

**Affiliations:** ^1^ Chitkara College of Pharmacy, Chitkara University, Punjab, India, Rajpura, Punjab India; ^2^ Chitkara College of Pharmacy, Chitkara University, Punjab India

## Abstract

**Background:**

Alzheimer’s disease is an advanced neurodegenerative illness that disturbs cognitive and behavior. One of the cores neuropathologic findings of AD is generation of hyper‐phosphorylated tau induced neurofibrillary tangles and Glycogen synthase kinase‐3β is the main kinase for the same. Tideglusib, a drug which is under second phase of clinical trial, impedes the GSK‐3β at therapeutically concentration and has been established to stop phosphorylation of tau.

**Method:**

Further, attempts have been made to conduct investigation and achieve advancements on Tideglusib analogues, utilizing scaffold morphing and the designed analogues were further evaluated using varied in silico techniques like pharmacokinetic, molecular docking and molecular simulations studies.

**Result:**

The designed molecules showed good interactions with the catalytic dyed residue (Asp228 & Asp32) of GSK‐3β having drug like properties. The stability of ligand‐protein complex was validated by using molecular dynamics studies in the time interval of 100ns.

**Conclusion:**

This design strategy could be used for the lead optimization of GSK‐3β Inhibitors and focusing attention on the exciting therapy could thus reap considerable clinical and economic rewards.

